# Calcination does not remove all carbon from colloidal nanocrystal assemblies

**DOI:** 10.1038/s41467-017-02267-9

**Published:** 2017-12-11

**Authors:** Pratyasha Mohapatra, Santosh Shaw, Deyny Mendivelso-Perez, Jonathan M. Bobbitt, Tiago F. Silva, Fabian Naab, Bin Yuan, Xinchun Tian, Emily A. Smith, Ludovico Cademartiri

**Affiliations:** 1 Department of Materials Science & Engineering, Iowa State University of Science and Technology, 2220 Hoover Hall, Ames, IA 50011 USA; 2 Department of Chemistry, Iowa State University of Science and Technology, 1605 Gilman Hall, Ames, IA 50011 USA; 3 Ames Laboratory, US Department of Energy, Ames, IA 50011 USA; 4 Instituto de Física da Universidade de São Paulo, Rua do Matão, trav. R 187, 05508-090 São Paulo, Brazil; 5 Michigan Ion Beam Laboratory, University of Michigan, Draper Road, Ann Arbor, MI 48109 USA; 6 Department of Chemical & Biological Engineering, Iowa State University of Science and Technology, Sweeney Hall, Ames, IA 50011 USA

## Abstract

Removing organics from hybrid nanostructures is a crucial step in many bottom-up materials fabrication approaches. It is usually assumed that calcination is an effective solution to this problem, especially for thin films. This assumption has led to its application in thousands of papers. We here show that this general assumption is incorrect by using a relevant and highly controlled model system consisting of thin films of ligand-capped ZrO_2_ nanocrystals. After calcination at 800 °C for 12 h, while Raman spectroscopy fails to detect the ligands after calcination, elastic backscattering spectrometry characterization demonstrates that ~18% of the original carbon atoms are still present in the film. By comparison plasma processing successfully removes the ligands. Our growth kinetic analysis shows that the calcined materials have significantly different interfacial properties than the plasma-processed counterparts. Calcination is not a reliable strategy for the production of single-phase all-inorganic materials from colloidal nanoparticles.

## Abstract (web summary)

Synthesis of all-inorganic nanomaterials often relies on organic templates, which are assumed to then be fully removed by calcination. Here, the authors use elastic backscattering spectroscopy to challenge this assumption, finding that calcination leaves behind considerable carbon content that can severely affect material function.

## Introduction

Organic ligands can control the growth of nanoparticles, prevent their agglomeration, and disperse them in solvents^1,2^. Nonetheless, they are often detrimental to the applications of colloidal nanocrystal assemblies (CNAs), especially to those requiring good transport properties (e.g. charge transport in solid state devices^3^, solar cells^4^, batteries^5^, fuel cells^6^) or those that require clean inorganic interfaces and surfaces (e.g., catalysis^7–9^).

Several methods have been used to remove organic ligands, e.g. solvent extraction, treatment with chemical stripping agents, UV-ozone treatment, calcination, and plasma processing. Each of these approaches have drawbacks: solvent extraction is generally effective on loosely adsorbed ligands and results in incomplete removal of carbon and minor grain growth^7^; chemical treatments are usually conducted in the liquid phase^10^ (which typically disrupts colloidal assemblies due to capillary forces) and do not completely remove all ligands^9,11^; UV-ozone treatment leads to oxidation of nanoparticles and incomplete removal of carbon^9,12^; chemical oxidation by ozone leads to incomplete carbon removal (53%) and can result in oxidation^13^; low-temperature plasmas O_2_ and air plasmas can lead to oxygen implantation or surface oxidation^14^, and the process takes a comparatively long time^15^, even though full etching can be accomplished in 6 h with optimized processing parameters.

Calcination at high temperatures in air remains the most common approach to remove ligands from CNAs, and, in general, to remove organics from nanostructured materials^9,16–23^. A search of the Google Scholar database yields ~60 thousand results for the keywords “template AND calcination”. While simple, this method has several limitations: high temperatures can coarsen the nanoparticles^24,25^, the oxidizing atmosphere can oxidize the nanoparticles (i.e., chalcogenides, oxides of partially oxidized metals)^26^, and exothermic reactions and the release of volatile byproducts can crack the CNAs^25^.

In spite of these limitations, calcination in oxidating environments is still widely used because it is equally widely assumed that it can fully remove organics from nanostructured hybrid materials in a relatively short time (recent reports claim full removal in as little as 60 s^8^). In the best cases the assumption is tested by Raman or Fourier-transform Infrared (FT-IR) spectroscopy to show the disappearance of C–H vibrational modes^8,21,27–30^, thermogravimetric analysis (TGA) to show mass loss^21,27,28,30,31^, or energy dispersive X-ray spectroscopy (EDX) to show the disappearance of the carbon signal^32^.

**Fig. 1:**
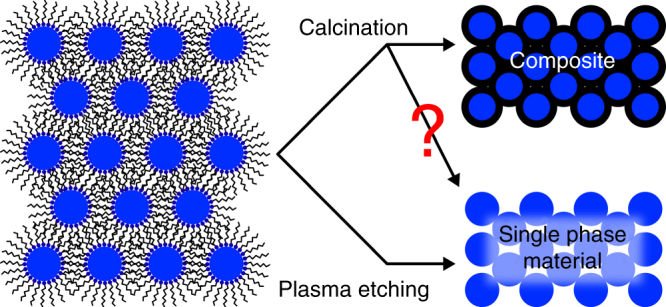
Difference between calcination and plasma processing. Schematic of the resulting microstructure after the ligand removal in colloidal nanoparticle assemblies (CNAs) by calcination and plasma processing

In this paper we demonstrate this assumption to be remarkably incorrect (Fig. 1), at least in the case of a model system consisting of 320 nm-thick films of 2.4 nm ZrO_2_ nanoparticles capped with trioctylphosphine oxide (TOPO). While Raman characterization indeed shows the disappearance of the C–H modes upon calcination, elastic backscattering spectrometry (EBS) characterization demonstrates that as much as 17.88% of the starting carbon atoms remains, even after calcination at 800 °C for 12 h. We compare these results with plasma etching on analogous samples which shows instead removal of 97% of the carbon atoms. Our findings suggest the troubling possibility that the calcination approaches ubiquitously used to convert hybrid nanostructures to inorganic crystalline phases (e.g., block-copolymer-templated mesoporous materials, aerogels, sol-gel coatings, inverse opals) yielded composites rather than the expected single-phase materials. It is especially troubling that the carbon phase left behind (most likely amorphous black carbon) is one of the hardest to detect reliably and quantitatively^33^, and yet it can severely affect properties. For example, we show here that calcined CNA displays a drastically accelerated grain growth kinetics (*E*_a_ = 30 vs. 111 kJ mol^−1^) when compared to plasma-processed CNAs^34^.

## Results

### Defining the model system

We chose ZrO_2_ nanoparticles as a model system for this study (2.4 nm ZrO_2_ capped with TOPO, cf. Supplementary Fig. 1—the polydispersity of the particles was 19% calculated as a 95% confidence interval). This phase is a refractory, which opens a wide range of calcination temperatures (300–800 °C) for study. The nanoparticles are dispersed in hexane and are stabilized in the tetragonal phase (cf. Supplementary Fig. 1b) at room temperature due to its lower surface energy than the thermodynamically stable monoclinic phase^35^. Spin coating of these hexane dispersions (nanoparticle concentration ~100 mg ml^−1^) form films (porosity ~20–25%^15^) that are especially resistant to cracking^36^.

### Calcination leaves carbon residue

The deposited CNAs were calcined in air (300, 400, 500 and 800 °C for 1, 3, 5 and 12 h, ramp up rate of 20 °C·min^−1^ and cool down rate of 2–3 °C·min^−1^). This choice of parameters was based on the typically reported calcination conditions for nanoparticles CNAs^19,24–26^. Control CNAs were exposed to oxygen plasma (7 W, 500 mTorr, 168 h) for comparison^15^. Optimized conditions allow for similarly effective etching in as little as 6 h.

**Fig. 2:**
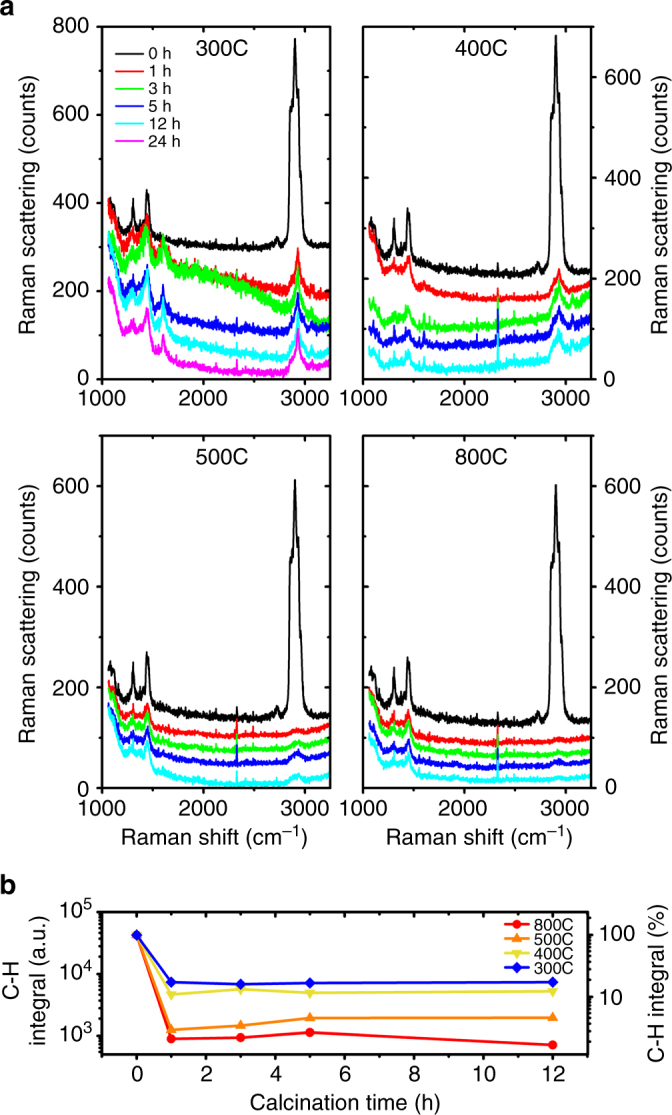
Effect of calcination on the C–H bond content.** a** Raman spectra of the ZrO_2_ CNAs before and after calcination at 300, 400, 500, and 800 °C for 1, 3, 5, and 12 h showing a reduction in the C–H content with increasing temperature. **b** The integral of the C–H Raman peaks for unprocessed and calcined CNAs with time, at different calcination temperatures. The plot shows a sharp decrease in the C–H content in the first 1 h of calcination followed by a plateau, at all temperatures

The Raman spectroscopy data from the calcined samples (Fig. 2) compared to the as-deposited films, provide an initial indication of the carbon removal. The five peaks between 2840 and 3000 cm^−1^ in the unprocessed CNA correspond to the C–H saturated bond stretching in the TOPO ligand. The peaks at 2847 and 2882 cm^−1^ represent the -CH_2_- bond stretching modes. The peaks at 2962 cm^−1^ may correspond to the -CH_3_ bond stretch. The peaks between 1100 and 1500 cm^−1^ may indicate other carbon bond stretching and are also characteristic of the ligand^37^.

Upon calcination, the height of the peaks between 2840 and 3000 cm^−1^ decreases significantly even after 1 h of calcination at 300 °C (Fig. 2a). The height of the peaks decreases with increasing temperature and almost vanishes at 800 °C. The increase in the time of calcination, results in a slow reduction in the peak heights. New peaks at 1601 and 3060 cm^−1^ appear after calcination at 300 °C and for longer times at 400 °C (Fig. 2a). They might originate from decomposition intermediates which decompose or oxidize at higher temperatures.

The integral of all C–H peaks (cf. Fig. 2b) shows a substantial reduction (as large as 98.3% for 800 °C, 12 h) upon calcination, especially in the first 1 h, followed by a plateau. While long calcination times do not seem to help significantly in removing the ligands, the plateau value does reduce with increasing temperatures. As the intensity of these peaks correlates with the number of -CH_2_- bonds, their reduction indicates the disappearance of these bonds and is in fact interpreted as a direct measure of carbon etching from thin films^8^.

**Fig. 3:**
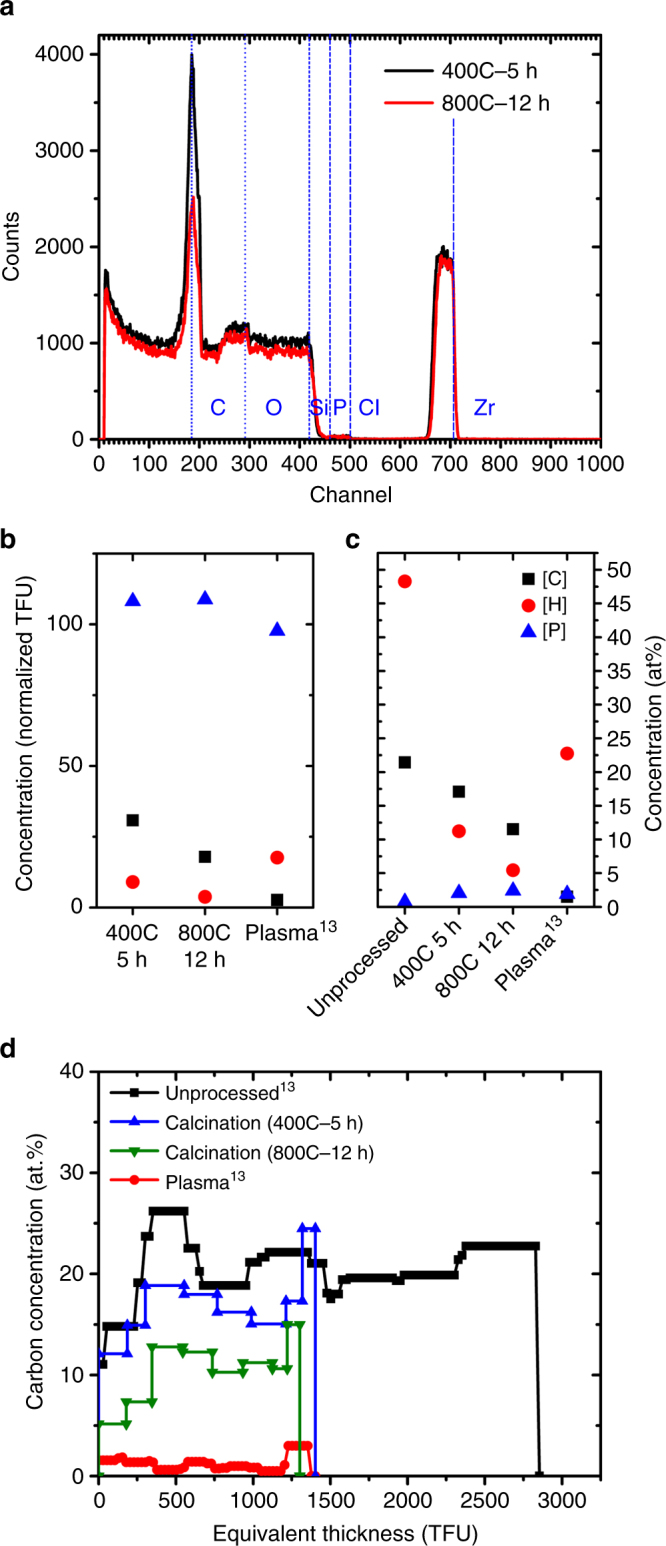
Chemical characterization after calcination and plasma processing. **a** Non-Rutherford elastic backscattering (EBS) spectra of the ZrO_2_ CNAs after calcination at 400 °C for 5 h and 800 °C for 12 h, showing significant carbon content (channel 185). **b** Concentration of C, H, and P in the films during calcination at 400 °C for 5 h, 800 °C for 12 h and plasma processing^13^, normalized with respect to concentration in unprocessed CNAs. **c** Concentration of C, H, and P in the unprocessed films, during calcination and plasma processing^13^, in at%. **d** Depth profile of the carbon concentration (in at.%) after calcination and plasma processing^13^

Since most thin films are calcined at temperatures ranging between 300–700 °C for times ranging between 3 and 4 h, we conducted EBS on samples calcined at 400 °C for 5 h and 800 °C at 12 h (Fig. 3a). The data clearly show a sharp peak at the 185th channel in the calcined samples, indicating significant concentrations of carbon. The area of the carbon peak is smaller for the 800 °C sample than for the 400 °C sample, which is consistent with the Raman spectroscopy results.

EBS uses a high energy (MeV) ion beam to measure the number of atoms of each element per unit area (in thin film units (TFU), i.e., 10^15^ atoms per cm^2^ or at.%). For this specific measurement EBS is superior to other elemental profiling techniques like time of flight-secondary ion mass spectrometry (TOF-SIMS), X-ray photoelectron spectroscopy (XPS), X-ray fluorescence, (XRF), EDX as it provides rapid, direct, accurate, and quantitative information on areal concentration of elements along with depth profiles, with a high sensitivity (<1 at.% detection limit for C), for films as thick as 500 nm^38^. Since EBS detects atoms rather than bonds (like Raman spectroscopy) or structure (like X-ray diffraction (XRD)), it is more reliable in the detection of total carbon content. Rayleigh backscattering, another ion beam analysis technique, has been rarely used to look at nanocrystal superlattices^39^.

Figure 3b, c show the EBS-derived concentrations of C, H, and P after calcination (this work) and plasma processing (data from Shaw et al.^15^). The plots represent the normalized TFU concentration of the elements with respect to unprocessed samples (Fig. 3b), and the atomic % (Fig. 3c). Calcination left behind 31% (400 °C, 5 h) and 18% (800 °C, 12 h) of the carbon atoms that were originally in the film. Hydrogen was instead much more severely reduced to 9 and 4% of the initial amount, respectively. Due to the effective removal of hydrogen, the concentration of carbon in the calcined samples only changes from 21 at.% to 17 at.% (400 °C, 5 h) and 12 at.% (800 °C, 12 h). A recently reported “flash” calcination approach^8^ was also not effective in this model system.

The difference in the etch rates for carbon and hydrogen during calcination indicates a loss of saturation of the organic fraction (the C/H ratio in the CNAs changes from 0.44 in the unprocessed film to 2.1 in the sample calcined at 800 °C), consistent with an insufficient supply of oxygen for a fully oxidative decomposition. These carbonization conditions usually yield highly crosslinked chars and black carbon that have reduced reactivity to oxygen. Ligand removal by calcination shares similarities with the thermal decomposition of polymers, during which various processes like chain scission, crosslinking, side chain elimination, and cyclization occur^40^. The elimination of smaller molecules also leads to the formation of bonds and crosslinking in the remaining chain. The residual crosslinked material is richer in carbon, higher in molecular weight, and is thus non-volatile. In the case of calcination, elimination of smaller hydrogenated molecules like methane, ethane, etc., through chain scission and formation of unsaturated bonds can explain the increased C:H ratio and the reduction of the C–H peaks.

Plasma processing, by comparison, reduces the number of carbon atoms by 97% (1.5 at.%). A smaller fraction of hydrogen is removed (82%), which is consistent with water adsorption. Phosphorus and zirconium (not shown) are unaffected by either treatments.

Figure 3d plots the depth profile of the carbon concentration (at.%) for calcined films (this work) compared to unprocessed and plasma-processed films (data from Shaw et al.^15^). The thickness of the CNA films is expressed in TFUs because EBS does not consider pores. Assuming a uniform density, the TFUs can be used as a measure of the CNA thickness. The plot indicates that carbon is uniformly distributed throughout the thickness of the films in all the calcination and plasma processing conditions. Our data do not support the existence of a gradient, which would be expected if diffusion inside the film is the kinetically limiting step. The higher carbon concentration at the surface of the films is due to adventitious carbon.

The diffusion of oxygen within these CNAs lies in the Knudsen regime^41^ even at 1 atm (the Knudsen number *K*_n_, which represents the ratio between the molecular mean free path and the pore size, lies between 142 and 265 for temperatures between 300 and 800 °C), due to their small pore size (~ 2 nm). The diffusivity inside the CNAs is orders of magnitude lower than in air (Knudsen diffusivity is estimated between 0.8×10^−7^ m^2^ s^−1^ at 300 °C and 1.2×10^−7^ m^2^ s^−1^ at 800 °C, instead of a molecular diffusivity between 0.4×10^−5^ m^2^ s^−1^ at 300 °C and 1.1×10^−5^ m^2^ s^−1^ at 800 °C^41^), but it does not limit the ligand removal due to the small thickness of the thin films. If all the carbon in the film were volatile, it would take as little as 10^−8^ s for it all to diffuse out at 298 K. These timescales are much shorter than the timescales of ligand etching or burnout in either plasma etching or calcination. Therefore, other factors are limiting the rate of ligand removal.

As will be discussed in a separate publication, the two key limitations to the etching kinetics are the chemical transformation of the organic matrix (i.e., crosslinking due to VUV radiation and reactions with radicals), and the formation of a boundary layer at the surface of the coating. The first mechanism is suggested on the basis of the observation that the rate of etching at intermediate and long etching times becomes proportional to the third power of the concentration of carbon in the film. The second mechanism is suggested on the basis of the accelerated rate of etching observed in the presence of pressure oscillations in the plasma chamber (as described by Cima and coworkers in the early 90s^42^).

### Calcination compromises the structural integrity of the films

**Fig. 4:**
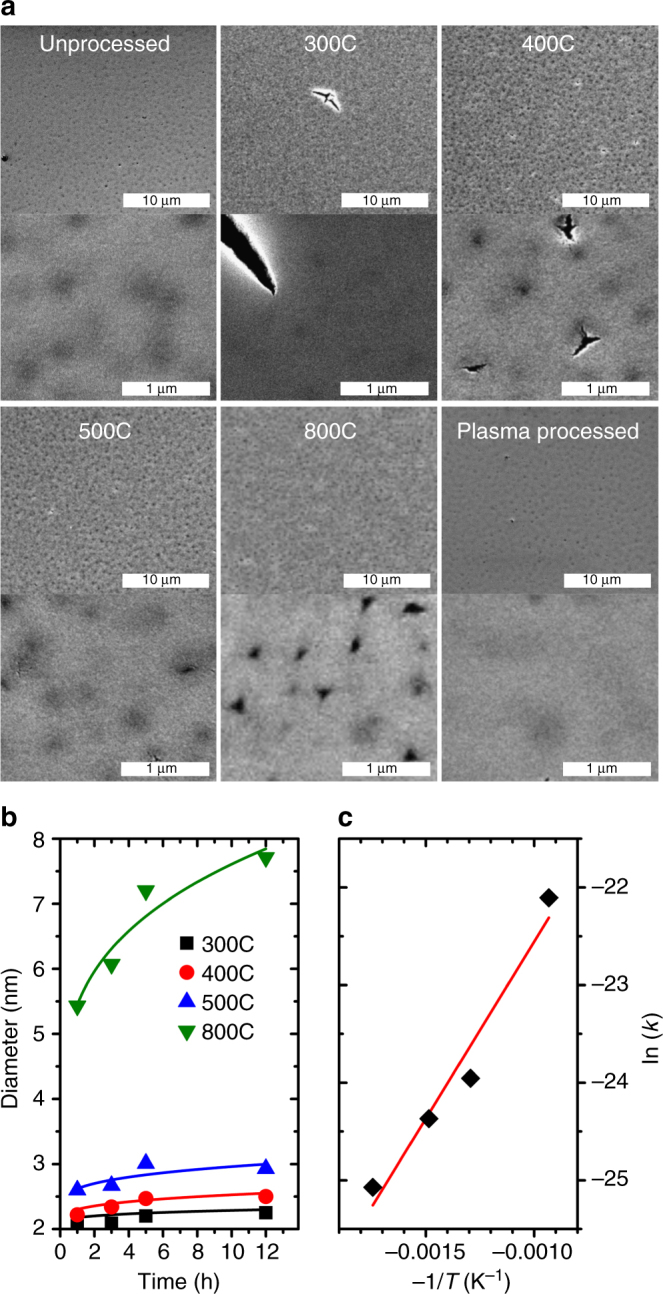
Surface characterization and growth kinetics during calcination. **a** SEM micrographs of the top surface of the unprocessed, calcined (12 h), and plasma-processed films. Plasma processing produces smooth films compared to calcination, which forms deep clefts, though the disordered structure of the film resists catastrophic cracking. **b** Crystallite size as a function of time at different calcination temperatures with Ostwald model fit curves. **c** Arrhenius plot of the rate constants of grain growth

One of issues of calcination is the accompanying rapid volume loss and gas formation which can damage the structure of the material^25^. The SEM images of the calcined films show what appear to be clefts on the surface of the films (cf. Fig. 4a). At higher temperatures, pits with extending microcracks can also be seen. Differently from previous reports^25^, even calcination for long periods (12 h) at temperatures (800 °C) did not cause extensive cracking in our model system. The cracks were not interconnected or intra-granular, and could therefore preserve some transport properties (like electrical conductivity) due to availability of a percolating path. The striking resistance to cracking in our films, compared to ordered CNAs, is consistent with the resistance to cracking upon plasma etching in disordered CNAs^36^. The plasma-processed film (Fig. 4a) is instead smooth and crack-free.

### Calcined films display an accelerated kinetics of grain growth

The XRD patterns of the calcined CNAs (cf. Supplementary Fig. 2) shows the diffraction peak associated with tetragonal ZrO_2_ growing sharper with increasing temperature and time, suggesting coarsening. No new phases are evident, suggesting that the remaining carbon is amorphous. Raman characterization showed no graphitization. The crystallite sizes are calculated by Scherrer analysis using the general expression $p = \frac{{K\lambda }}{{b{\times}{\mathrm{cos}}(\theta )}}$ where *p* is the “true size” of the crystallites (defined as cube root of the crystallite volume), *K* is the Scherrer constant whose value depend on crystallite shape, *b* is the breadth of the peak, *λ* is the X-ray wavelength, and *θ* is the diffraction angle^43^. The formula was corrected to account for the spherical shape of the particles (*K* = 0.829 and diameter = (6/π)^1/3^·*p*) as discussed in a prior publication^44^. At all temperatures, the crystallite sizes steeply increase in the initial time periods followed by a plateau at longer times (Fig. 4b).

An Ostwald ripening model^45^ describes our experimental data better than other models^46–48^, while having fewer floating parameters. The model describes the growth kinetic as *D*(*t*) = *D*(*t* = 0) + *k*×*t* ^1/*n*^, where *t* is time, *D(t)* is the time-dependent particle size, *k* is the temperature-dependent rate constant and *n* is the growth parameter that indicates the rate limiting process in the growth kinetics^48^. The calcined samples yielded a *n* value of 3.6 which is significantly different from the value reported for TOPO-capped ZrO_2_ nanoparticles that have been sintered only after complete ligand removal by plasma processing (2.28 ± 0.27)^34^. This analysis suggest that the rate of diffusion of the ions at the particle/matrix boundary regulates growth kinetics in plasma-processed samples, while the rate of dissolution of the surface atoms limits growth in calcined films. The rate constants of calcined samples show an Arrhenius dependence on temperature (Fig. 4c). The activation energy of grain growth of the calcined CNAs (30 kJ mol^−1^) is nearly four time smaller than the one measured in plasma-processed samples (111 kJ mol^−1^ ^34^). Lower activation energies suggest earlier growth/coarsening during calcination in comparison to the plasma-processed samples. Since grain growth is a surface-dominated process, the significant reduction in the activation energy suggests a significant change in the interface composition resulting from the incomplete oxidation of the ligands.

While our results apply quantitatively to our model system, it is likely that qualitatively similar results occur in similarly structured materials, with some possible exceptions, e.g., materials in which oxidative decomposition of organics is catalytically accelerated (e.g., Pt, Pd)^8,28^.

## Methods

### Particle synthesis

The synthesis of TOPO-capped ZrO_2_ nanocrystals as described in our earlier paper^36^. Eighty grams of TOPO was taken in a three-neck round bottom flask with a condenser attached to the middle neck and septa on the side necks. The TOPO was liquefied under argon and then degassed under vacuum at 80 °C for 30 mins with constant stirring. 20 mmol of zirconium (IV) chloride and 16 mmol of zirconium (IV) isopropyl alcohol complex was added to the liquefied TOPO and temperature of the mixture was raised to 340 °C under an argon flow. After holding the reaction mixture for 2.5 h at 340 °C under argon blanket, the temperature was lowered to 80 °C and then diluted with toluene in a 1:1 ratio. The nanoparticles were then cleaned with acetone by centrifugation and dispersed in hexane.

### CNA deposition and processing

Spincoated CNAs were calcined in a furnace, with static air. The heating rate was maintained at 20 °C min^−1^. After the calcining time, the samples were annealed to room temperature in the furnace at ~2 °C min^−1^.

### X-ray diffraction

Powder X-ray diffraction (XRD) was performed with Siemens D500 X-ray diffractometer at Materials Analysis and Research Laboratory (MARL) at Iowa State University. The diffraction pattern of as-synthesized ZrO_2_ nanoparticles was collected from drop casted film on a zero diffraction plate, whereas, pattern from plasma-processed sample was collected from CNA spincoated on silicon substrate. XRD patterns were collected in 2θ range 20–60° by using 0.15DS, 0.05° steps, and 3 s per step. For nanoparticle size analysis, diffraction pattern was collected in slow scan mode (0.03° steps and 20 s integration) to improve peak-to-noise ratio. Accurate measurement of nanoparticles size using Scherrer equation was done following the procedure reported^44^.

### Raman spectroscopy

Raman spectroscopy measurements were performed using an XploRa Plus confocal Raman microscope (Horiba Scientific/JY, France) equipped with a 532-nm laser excitation source (11 mW at the sample). Raman spectra were collected at three locations from the center of each film under ambient laboratory conditions using a 50× air objective (Olympus, LMPlanFL) with a 0.5 numerical aperture. The spectra were collected from 900 to 3300 cm^−1^ with a 1200 grooves/mm grating. Reported spectra were an average of 3 measurements, with a 120 s acquisition time for each spectrum.

### Scanning electron microscopy

Scanning electron microscopy was performed with FEI quanta 250 field-emission SEM at both MARL and Department of Materials Science and Engineering at Iowa State University. Samples with poor conductivity were sputtered coated with 5 nm iridium before imaging. Imaging was done in secondary electron mode under high vacuum at 8 keV with beam spot size of 2.5.

### Transmission electron microscopy/scanning transmission electron microscopy

Transmission electron microscopy (TEM) and high resolution TEM images were obtained using 2007 JEOL 2100 200 kV STEM in TEM mode, operating at 200 kV. This STEM is located at Microscopy and NanoImaging facility, Iowa State University and equipped with a Thermo Fisher Noran System 6 X-ray microanalysis system. Samples for TEM analysis were prepared by evaporating drops of dilute nanocrystals dispersion at room temperature, on a carbon-coated copper grid. Energy dispersive X-ray (EDX) analysis were performed in STEM mode. Samples for EDX characterization were prepared by scraping off flakes of CNAs from the substrate using a sharp blade, and then attaching them on a carbon-coated TEM grid.

### Ion beam analysis

All ion beam analysis measurements were carried out at the Michigan Ion Beam Laboratory^49^ at the University of Michigan with the 1.7 MV Tandetron accelerator.

The elemental analysis of the samples throughout the film depth was determined by combining non-Rutherford elastic backscattering spectrometry (EBS) and elastic recoil detection (ERD) using a helium beam.

The EBS and ERD spectra were taken simultaneously at two different energies for each sample. A 3040-keV He^++^ beam was used for sensitivity to the oxygen signal through the EBS resonance at 3038.1 keV^50^. Similarly, a 4290-keV He^++^ beam was used for sensitivity to the carbon signal through the EBS resonance at 4265 keV^51^.

The samples were mounted on a sample plate on the 5-axis goniometer of the 2 MV Tandem accelerator. The scattering angle of the EBS detector was 170° and of the ERD detector 30°. For each measurement the beam incident angle was 70°. The filter in front of the ERD detector was a 24 µm thick foil of Kapton (C_22_H_10_O_5_N_2_; density of 1.42 g cm^2^). The beam current on the samples during these measurements was ~20 nA with a beam spot of 1.5 mm by 1.5 mm.

The spectra evaluation followed a self-consistent approach enabled by MultiSIMNRA^52^ that uses SIMNRA^53^ code as engine to calculate the simulated spectra. The MultiSIMNRA code enables the combination of multiple spectra by the optimization of an objective function calculated for all spectra. The final depth profile emerges from the optimization algorithm as the model that best describes all experimental data simultaneously. The main advantage of the self-consistent approach is that the information contained in one spectra plays as boundary condition during the optimization of all the others.

All simulations used the SRIM stopping power^54^ for energy loss calculations and SigmaCalc^55^ scattering cross-sections of Helium in Oxygen and Carbon. For some reason that stays unclear, a better agreement to the experimental data was obtained using the scattering cross-section of Helium on Silicon provided in ref. ^56^ rather than by SigmaCalc. Andersen screening function to Rutherford cross-sections^57^ and the empirical model by Yang for the energy loss straggling calculations^58^ were adopted. The geometrical straggling was taken into account for all simulations, and for the ERD simulations the multiple scattering was also calculated.

### Data availability

The data that support the findings of this study are available in the article, the Supplementary Information, and from the corresponding author upon reasonable request.

## Electronic supplementary material

Supplementary Information
