# Supplementary material for: Calcination does not remove all carbon from colloidal nanocrystal assemblies
Source: Nat Commun. 2017 Dec 11;8:2038. doi: 10.1038/s41467-017-02267-9 (PMC5725572; doi:10.1038/s41467-017-02267-9)
Supplement: Supplementary file 1 — Supplementary Information [file 41467_2017_2267_MOESM1_ESM.pdf]

SUPPLEMENTARY FIGURES

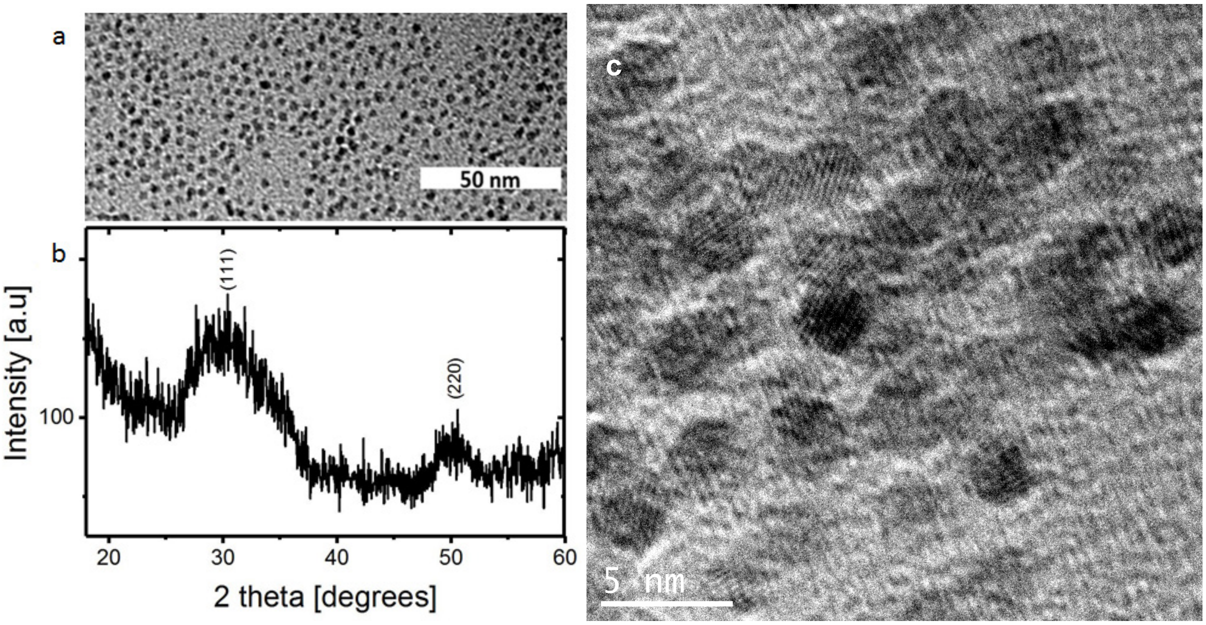


**Supplementary Figure 1. Morphological and phase characterization of the colloidal zirconia nanoparticles.** **(a)** TEM micrographs of the TOPO capped zirconia nanoparticles dispersed in hexane **(b)** XRD spectrum of the unprocessed $ZrO_2$ nanoparticles, showing predominantly tetragonal phase. **(c)** Representative TEM of $ZrO_2$ nanoparticle showing single crystalline structure.

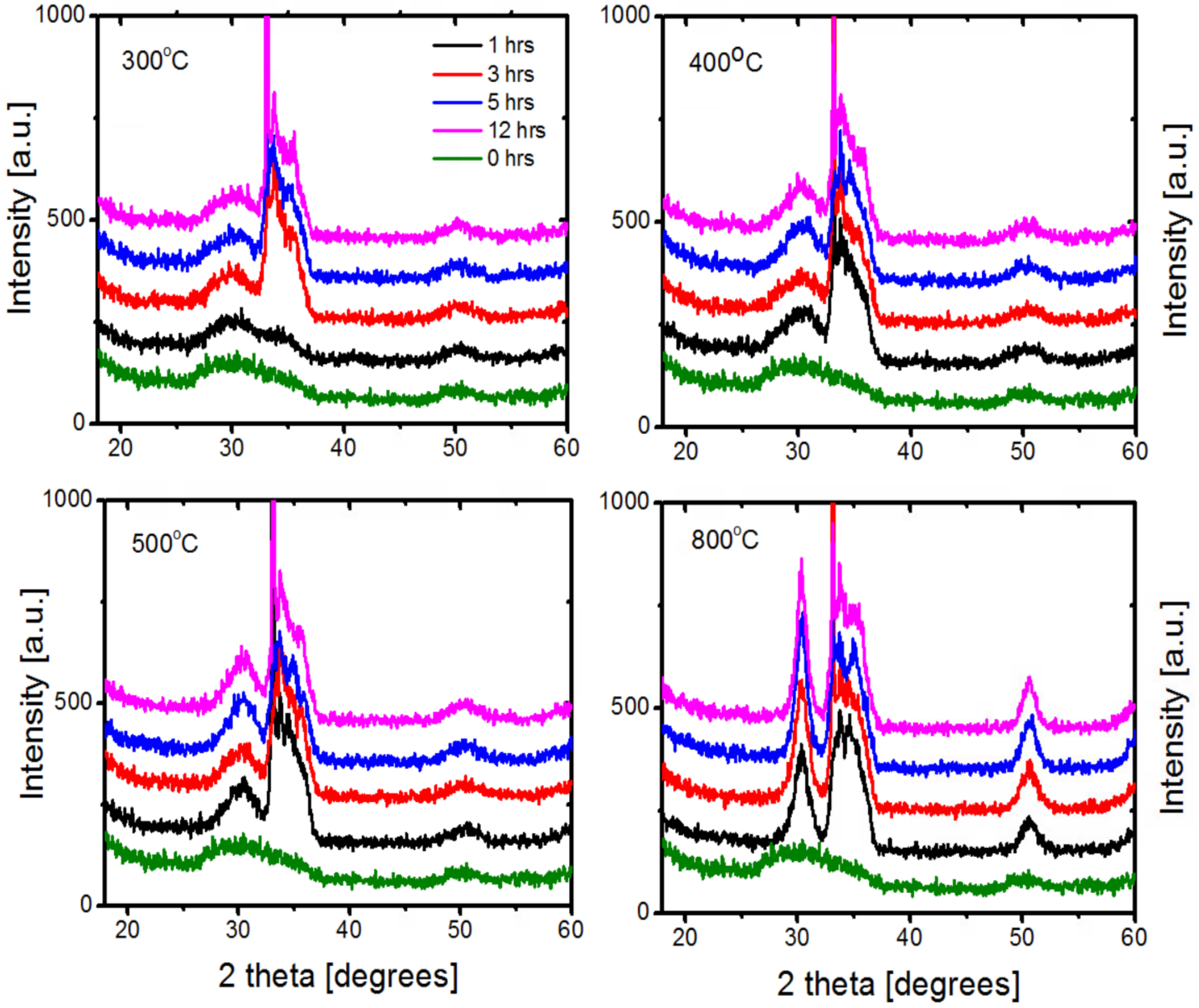


**Supplementary Figure 2. XRD spectra of the CNAs at different calcination conditions.** The plots show an increase in the tetragonal zirconia peak with the increase in temperature and time of calcination (indicating an increase in the crystallite size) and no appearance of new phases. The sharp peaks at 33-38° are due to the Si substrate.

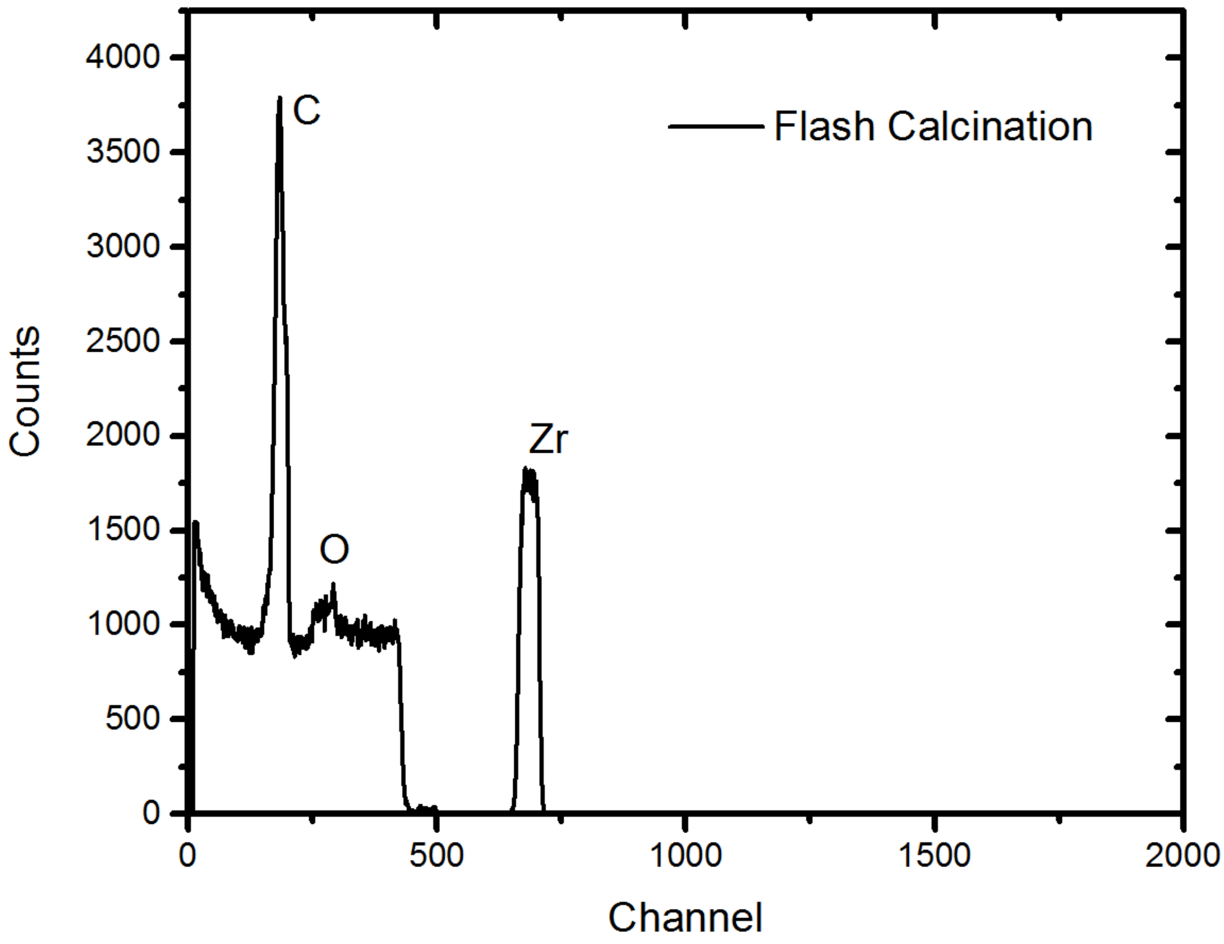


**Supplementary Figure 3. EBS spectrum of $ZrO_2$ nanoparticle CNAs.** The CNAs are calcined according to the approach detailed by Cargnello et al.[1]. The remaining carbon is clearly shown, while Raman spectroscopy failed to detect any remaining C-H bonds.

## SUPPLEMENTARY REFERENCES

1. Cargnello, M. et. al. Efficient removal of organic ligands from supported nanocrystals by fast thermal annealing enables catalytic studies on well-defined active phases. *J. Am. Chem. Soc.* **137,** 6906-6911 (2015).
